# Autoimmune Diseases and Mycobacterial Infection

**DOI:** 10.3390/diseases14030099

**Published:** 2026-03-07

**Authors:** Abraham Chorbajian, Ira Glassman, Akhila Swarna, Manvita Mareboina, Po-En Chen, Jammal Abu-Khazneh, Jiayan Tan, Surbi Dayal, Kian Yazdan, Bianca Urness, Vishwanath Venketaraman

**Affiliations:** 1 College of Osteopathic Medicine, Western University of Health Sciences, Pomona, CA 91766, USA; abraham.chorbajian@westernu.edu (A.C.); ira.glassman@westernu.edu (I.G.); akhila.swarna@westernu.edu (A.S.); poen.chen@westernu.edu (P.-E.C.); jammal.abukhazneh@westernu.edu (J.A.-K.); jiayan.tan@westernu.edu (J.T.); surbi.dayal@westernu.edu (S.D.); kian.yazdan@westernu.edu (K.Y.); bianca.urness@westernu.edu (B.U.); 2 Penn State College of Medicine, Hershey, PA 17033, USA; manvita.mareboina@gmail.com

**Keywords:** autoimmune disease, mycobacteria, rheumatoid arthritis, type 1 diabetes mellitus, systemic lupus erythematous, multiple sclerosis, Crohn’s disease

## Abstract

Background/Objectives: Mycobacterial infections and autoimmune diseases affect many worldwide, and growing evidence suggests that there is a bidirectional relationship. This review examines mechanisms by which various autoimmune diseases predispose patients to mycobacterial infections, and vice versa. Methods: We conducted a PubMed/MEDLINE search using the keywords “mycobacterium” and the names of the autoimmune conditions to identify relevant papers. Results: Rheumatoid arthritis therapies, especially TNF-α inhibitors, raise tuberculosis (TB) and non-tuberculous mycobacteria (NTM) risk. Type 1 diabetes features impaired cell-mediated immunity and macrophage dysfunction, with evidence for *Mycobacterium avium* subspecies *paratuberculosis* (MAP) mimicry involving HSP65–GAD65. In systemic lupus erythematosus, immune dysregulation plus corticosteroids and cytotoxins elevates TB and NTM risk, amplified in endemic settings. In multiple sclerosis, heightened TLR2/4/9 signaling agents that inhibit pyrimidine synthesis may increase IL-10 and reduce antimycobacterial immunity. Crohn’s disease shows genetic susceptibility (e.g., NOD2 variants) and MAP detection, supporting impaired clearance of intracellular mycobacteria. Conclusions: Overall, evidence supports a bidirectional relationship: mycobacterial antigens can initiate or amplify autoimmunity via molecular mimicry and chronic stimulation, while autoimmune biology and iatrogenic immunosuppression increase susceptibility to infection. Implications include latent TB screening before immunosuppression, attention to local epidemiology, and vigilance for NTM. Research priorities include prospective cohorts, mechanistic studies of mimicry and NOD2–TLR pathways, safety registries, and trials of screening and prophylaxis.

## 1. Introduction

Mycobacterial infections are one of the leading causes of death and disease worldwide. In 2025, *Mycobacterium tuberculosis* (*M. tb*) caused an estimated 10.7 million infections and 1.23 million deaths, while non-tuberculous mycobacterial (NTM) infections continue to rise in many parts of the world [1,2].

An acid-fast bacterium that uses humans as its reservoir, *M. tb* tends to remain dormant inside granulomas of immunocompetent individuals, leading to latent tuberculosis infection (LTBI) that can become reactivated and cause disease upon weakening of the immune system; recent estimates suggest that the global burden of LTBI is approximately 1.7 billion people, nearly one-fourth of the global population [3]. Interestingly, the incidence rate and the estimated number of infections and deaths from *M. tb* have increased since the onset of the COVID-19 pandemic, reversing many years of decline. Current models are even suggesting further increases in *M. tb* deaths and incidence rates, possibly restoring its position as the single leading cause of death globally by infection [2]. Aside from death, these increasing rates of infection can have profound consequences on various other facets of human health, including autoimmune diseases.

Although historically relatively uncommon, the incidence of autoimmune diseases appears to be on the rise, especially in industrialized countries [4,5,6]. While a handful of hypotheses have been formulated to explain this phenomenon, the development of autoimmune diseases is multifactorial and dependent upon a host of genetic, immunological, hormonal, and environmental factors collectively referred to as the “mosaic of autoimmunity” [7,8]. With special regard to environmental factors, it has long been established that infections can induce or cause autoimmune diseases via aberrant activation of the immune system in susceptible individuals through mechanisms such as molecular mimicry, epitope spreading, bystander activation, persistent infection, and polyclonal activation, among others [6,7,8,9]. In fact, several studies have reported increased quantities of various autoantibodies [10,11,12,13,14] as well as alterations in the levels of specific B and T lymphocytes in tuberculosis (TB) patients; these are both consistent with autoimmune pathogenesis [15,16,17,18]. Interestingly, many of these autoantibodies decreased or even normalized after TB treatment [10,13,14,19]. Conversely, increased antibodies against certain mycobacterial species, such as *Mycobacterium avium* subsp. *paratuberculosis* (MAP), have been found in patients with autoimmune conditions [20,21,22].

On the other hand, researchers have repeatedly found that patients with autoimmune diseases have an increased susceptibility to developing infections, which may be attributable to the immunosuppressive therapies they undergo as part of their disease treatment. However, the increased susceptibility may also be due to the genetics of a particular autoimmune disease and its implications in immunodeficiency, as well as the immune dysregulation responsible for the autoimmune diseases. This can include lymphopenia, altered T cell responses, complement deficiency, and more [9,23,24,25]. Therefore, autoimmune diseases and infections have a bidirectional relationship.

Furthermore, the notion of autoimmune diseases correlating to certain geographical areas has prevailed for decades, with the reported north–south gradient in the early 2000s illustrating that the incidence of autoimmune diseases decreases from north to south in the Northern Hemisphere and opposingly south to north in the Southern Hemisphere; this pattern still holds today [6,8]. Conversely, the geographical distribution of multiple autoimmune diseases appears to be the opposite of TB among other infections; however, this can be attributed to several distinct factors, such as climate/sun exposure (vitamin D levels) and socioeconomic status (lower SES is associated with poorer sanitation) [6,8].

In this review, we aim to outline the susceptibility to mycobacterial infections, especially *M. tb* and *M. avium*, in patients that suffer from autoimmune diseases, particularly rheumatoid arthritis (RA), type 1 diabetes mellitus (T1DM), systemic lupus erythematous (SLE), multiple sclerosis (MS), and Crohn’s disease, as these are among some of the most prevalent autoimmune diseases worldwide, many of which have been associated with an increased risk of *M. tb* [26,27,28]. In addition, we will explore the role that infection with the various mycobacterial species plays in the development of autoimmune disease and any regional associations between the two that may exist.

## 2. Materials and Methods

We searched PubMed/MEDLINE for studies pertaining to mycobacterium infections and autoimmune diseases and examined articles published from 1970 to 2025. The final search was conducted on 25 October 2025 and included the following keywords: “mycobacterium”, “rheumatoid arthritis”, “type 1 diabetes mellitus”, “systemic lupus erythematosus”, “multiple sclerosis”, and “Crohn’s disease”. Studies were included if they discussed the pathology of the autoimmune disease and its relevance to mycobacterium infections.

## 3. Results

### 3.1. Rheumatoid Arthritis

RA is a systemic autoimmune disease that affects 0.5–1.0% of the adult population [29]. RA is characterized by joint pain and swelling, localized in the hands, feet, and wrists, resulting in progressive bone and cartilage damage [30]. Synovial inflammation and bone damage are hallmarks of RA and result in the proliferation of immune cells such as B and T lymphocytes, neutrophils, and synovial fibroblasts. Lastly, the increased proliferation of immune cells makes RA a chronic inflammatory disorder, which increases the patient’s susceptibility to infections. In vitro and in vivo studies display the pathogenic and biological link between mycobacterial infections and the development of RA. For instance, Liao et al. found that compared to the general population, RA patients are 2.28 and 6.24 times more likely to be infected by *M. tb* [31]. Patients with RA have an increased risk of mycobacterial infections due to immune disturbances caused by the disease itself, as well as the immunosuppression from medication used to treat RA [32].

RA pathogenesis involves pro-inflammatory cytokines such as tumor necrosis factor-alpha (TNF-α), IL-1, IL-6, and immune cells; therefore, biological agents targeting these cytokines and immune cells have been developed to decrease the prevalence of disease in RA patients [33]. Treatment for RA involves disease-modifying anti-rheumatic drugs (DMARDs), including tumor necrosis factor inhibitors (TNFIs), which reduce joint damage and produce better health outcomes [34]. While DMARDs are useful in RA immunomodulation, they increase the risk of TB infections via immunosuppressive activity [35].

Methotrexate, azathioprine, cyclophosphamide, cyclosporine, or corticosteroids are common medications used to treat opportunistic and non-opportunistic infections; however, the use of these drugs has been accompanied by a rise in the rate of new and relapsing cases of TB in the last decade [36]. In addition, TNF-α inhibitors have now become increasingly popular for the treatment of rheumatic diseases. In TNFI therapy, TNF-α, a pro-inflammatory cytokine that causes synovitis, is blocked by the drug to reduce inflammation signaling in RA [37]. However, following TNFI therapy, the risk of opportunistic infections such as TB increases. In the first TNFI randomized control trial, one case of *M. tb* was reported, and since then, evidence supporting the link between TNFI and the increased risk of TB has been accumulating [38]. For instance, a study with a cohort of 56,269 older adults with RA reported current users of anti-TNF agents being at an increased risk of both TB and NTM [39]. In addition, specific TNFIs such as infliximab (INF) and adalimumab (ADA) (both monoclonal antibodies) have a higher risk of causing *M. tb* infection than etanercept (ETA), a soluble TNF receptor [40]. Overall, patients with RA are at an increasing risk of developing TB due to immunosuppression from medication used to treat the disease.

### 3.2. Type 1 Diabetes Mellitus

T1DM is a chronic metabolic disorder caused by autoimmune destruction of pancreatic β-islet cells, leading to insulin deficiency and subsequent hyperglycemia. Although its precise pathogenesis remains incompletely understood, current evidence suggests that T cell-mediated β cell destruction arises from a complex interplay of genetic susceptibility and environmental triggers [41]. *M. tb* and NTM infections pose a substantial risk for individuals with compromised immune systems, leading to considerable morbidity and mortality within this patient population [42]. *Mycobacterium* sp. is ubiquitously found throughout environments shared with humans and domesticated animals, leading to indefinite exposure [43]. Given the rising global prevalence of T1DM, investigating the relationship and potential overlap between this disease and mycobacterial infections represents an important and emerging area of research.

Annually, there is an estimated 9.6 million patients newly infected with active TB; moreover, 1 million within the population have both TB and T1DM [44]. A person who has diabetes is approximately three times more likely to develop TB compared to someone without diabetes, resulting in adverse treatment outcomes, including death [45]. In a cross-sectional study performed by Raghuraman et al., the prevalence of diabetes and TB for patients within India was 29% [46]. Additionally, a prospective study by Restrepo et al. found a 39% prevalence of diabetes among TB patients within Texas, and 36% in Mexico [47]. Alternatively, a cross-sectional analysis performed by Olayinka et al. concluded only a 5.7% prevalence finding between TB and diabetic patients within Nigeria, and 6% in Lagos Sate [48]. The notable difference in prevalence reported between the different groups could stem from the lower national prevalence of diabetes within Africa when compared to North America [6,8,49]. Furthermore, Olayinka et al.’s study was conducted within a hospital setting, which may not accurately reflect the true prevalence within the community [48].

Insulin plays a critical role in modulating immune function; therefore, the insulin deficiency characteristic of T1DM can impair cell-mediated immunity, leading to reduced activity of cytotoxic CD8^+^ T cells and helper CD4^+^ T cells [47,50]. Insulin deficiencies lead to an imbalance of pro-inflammatory and anti-inflammatory cytokines responsible for maintaining a balanced and regulated immune response to pathogens [51,52]. This leads to further immune dysregulation, particularly involving impaired cell-mediated immunity, altered cytokine production, and defective and reduced macrophage function [47,51,52,53]. Studies have shown that elevated glycation could inhibit the cytokines IL-10, interferon gamma (IFN-γ), and TNF-α. Moreover, glycation also hinders the expression of class I major histocompatibility complex (MHC) on myeloid cells, repressing cell immunity [54]. Ultimately, reducing T cell function prevents protection from intracellular pathogens.

Macrophages play an important role in the early immune response against foreign pathogens such as mycobacterial infections. However, in T1DM, macrophages commonly have impaired phagocytic activity, which prevents degradation of intracellular pathogens [53]. Saiki et al. discovered that in diabetic mice, macrophages had a 90% decrease in phagocytic activity compared to control mice [55]. Wang et al. proved that patients with TB had fewer activated alveolar macrophages and reduced hydrogen peroxide production in diabetic individuals [56]. Additionally, T cell production of IFN-γ is reduced, which is responsible for nitric-oxide-dependent intracellular degradation within macrophages. Experiments involving a murine model system illustrated a reduced level of IFN-γ in diabetic mice [57]. Evidently, multiple studies have shown the reduced phagocytic activity of macrophages, illustrating why patients with T1DM are even more susceptible to pathogens.

Despite multiple proposed mechanisms of increased susceptibility to mycobacterial infections in diabetic patients, recent findings suggest that MAP can induce T1DM through molecular mimicry [58]. T1DM is characterized by increased levels of autoimmune targeting of self-antigens, specifically heat shock protein (HSP), glutamic acid decarboxylase (GAD), and insulinoma-associated protein-2 (IA-2) [59]. The GAD65 isoform is the rate-limiting enzyme responsible for the conversion of glutamic acid to γ-amino butyric acid (GABA) and is uniquely expressed in β-islet cells [60]. Heat shock protein 65 (HSP65) is specifically expressed in mycobacteria and has overlapping amino acid homology with GAD65 [61]. Mehra et al. found that nearly 70% of T1DM patients have antibodies directed towards GAD65, while only 4% of non-diabetic individuals produced antibodies [62]. Additionally, Santos et al. demonstrated that mycobacterial DNA vaccines protecting mycobacterial HSP65 prevent diabetes within mouse models [63]. These findings collaboratively suggest that molecular mimicry could play a fundamental role in T1DM destruction of β-islet cells when exposed to MAP.

Multiple studies have indicated that T1DM is associated with impaired immune function, including compromised cell-mediated immunity, altered cytokine production, and defective macrophage function, thereby increasing one’s vulnerability to mycobacterial infection [64].

### 3.3. Systemic Lupus Erythematosus

SLE is a chronic systemic disease in which the body’s immune system attacks its own tissues and organs via antinuclear antibodies, anti-dsDNA antibodies, anti-Smith antibodies, and anti-Ro antibodies [65]. This dysregulated autoimmune process leads to inflammation most commonly in the skin and joints and can lead to further tissue damage in the kidneys, brain, lungs, and cardiovascular system. This disease primarily affects women in reproductive years (between the age of 18 and 35) and is more prevalent in Hispanic, African American, and Asian patient populations [66,67]. Due to their compromised immune systems, patients with SLE are at a greater risk of developing infections, especially mycobacterial infections, with significant effects on pulmonary function and disease progression [68]. Retroviruses and epigenetic changes caused by infections like that of mycobacteria are thought to aid the pathogenetic mechanisms operating in SLE [69]. Recent work by Wen et al. further elucidates this connection, showing that dysregulated activation of Toll-like receptors 7 and 9 (TLR7/9) in SLE promotes excessive B cell activation and autoantibody production, amplifying systemic inflammation and impairing pathogen defense [70]. In addition, immunosuppressive therapy, particularly prolonged or high-dose corticosteroids, further increases TB risk in SLE by causing lymphopenia and blunting Th1-mediated macrophage activation (reduced IFN-γ/TNF-α signaling), compromising containment of mycobacterial infection [71].

SLE is unique in that its symptoms can present variably, rendering case studies and single-center retrospective studies as the main ways mycobacterial infections are documented in patients with SLE. For instance, a case study of *Mycobacterium kansasii* infection in the foot joint of a 46-year-old woman with SLE evaluated the impact of infection with the bacteria in the joint, bone, and periarticular structures. The patient’s diagnosis was made via positive culture from degenerative tissue and histological analysis, with researchers proposing an increased risk of co-infection in the setting of rheumatic diseases [72]. Similarly, another case followed a 19-year-old female who presented with persistent fever, dry cough, and polyarthralgia despite empiric antibiotic and steroid therapy. Sample cultures from endobronchial ultrasound-guided transbronchial needle aspiration of mediastinal lymph nodes revealed *Mycobacterium kansasii*, antinuclear antibodies, and lupus anticoagulants. The diagnosis of *Mycobacterium kansasii* infection associated with SLE was made, indicating mycobacterial infections as a possible infectious trigger of autoimmunity [73].

Many studies on the association between SLE and TB infections have occurred in locales with a higher prevalence of TB infections. In a single-center case study in Indonesia, patients with SLE who contracted TB had greater rates of developing lupus nephritis from prolonged corticosteroid use than patients who did not contract TB infection [74]. This conclusion was also supported by another cohort in West Java, Indonesia, that found that a history of TB, high-dose corticosteroid use, and younger age of SLE diagnosis are key factors that increase the risk of TB infection in patients with SLE [75]. Similarly, a retroactive study evaluating a cohort of 725 SLE patients in Hong Kong showed that SLE disease duration was found to be the only independent predictive factor for NTM infections [76].

When evaluating the relationship between TB infection and SLE in Taiwan from January 2000 to December 2008, an average annual incidence rate of 8.1 per 100,000 was found and TB posed the greatest risk for SLE patients [77]. Comparably, in a cohort of 70 SLE patients diagnosed over a 2-year period, 14 patients were found to have confirmed antecedent TB (20.0%), which was 40 times higher than the prevalence of TB in the local population [78]. Unlike other studies, however, TB medication and treatment were ruled out as the causative factor in precipitating SLE in these patients via an analysis of anti-histone antibodies of SLE patients with and without antecedent anti-TB treatment [78].

While the cohorts discussed were mainly in West Asia, where there are larger TB-endemic countries such as India and China, a study investigating the frequency of TB infection in patients with SLE in Santa Clara Valley Medical Center in Northern California found that 25% of those patients had latent or active TB infection [79]. Many of the patients at Santa Clara Valley Medical Center included a significant immigrant population of 45% of the cohort from Mexico, which is a locale of high incidence of active TB [80]. With immigration increasing worldwide, it is suggested that the incidence and prevalence of TB could increase and thus pose a risk of infection for patients with SLE.

### 3.4. Multiple Sclerosis

MS is a chronic autoimmune disease that affects the central nervous system, leading to a range of neurological symptoms and disabilities. Several studies have shown a strong association between MS and *M. tb*, but the specific mechanism remains unclear. Cossu et al. suggested that cell-mediated immunity protects non-MS, healthy individuals against mycobacterial infection, such as pulmonary TB [81]. This could be due to the overlapping roles of TLR2, TLR4, and TLR9 in immune responses during MS and TB.

Toll-like receptors (TLRs) have been shown to play an essential role in combating invading bacteria, and their activation leads to the release of cytokines or chemokines that mediate effective adaptive immune responses [82]. There have been recent studies reporting an increased expression of TLR2, TLR4, and TLR9 in MS patients [81]. These TLRs interact with various immune cells, recognize mycobacterial components, and activate autoreactive T cells, thus contributing to MS [81,83].

While TLRs have demonstrated protective effects against mycobacterial infection, dysregulation of this signaling pathway can have the opposite outcome [49]. For instance, the activation of TLR2 has been shown to contribute to the elimination of *M. tb* in mouse macrophages through a nitric-oxide-dependent killing pathway [84]. However, excessive TLR activation can lead to inflammation and a suboptimal antimycobacterial immune response [49,85]. Persistent inflammation that disrupts the normal immune system can enhance *M. tb* risk by providing a favorable environment for mycobacterial growth. Specifically, overstimulation of immune cells can impair phagocytosis and reduce antimicrobial activity, reducing the ability to combat mycobacterial infection [86,87].

Another study suggested that the treatment of MS enhances risk of TB. Gozubatik-Celik et al. presented a case where a patient was diagnosed with TB after teriflunomide therapy, an immunomodulator that treats MS by inhibiting the dihydroorotate dehydrogenase enzyme, which is important for pyrimidine synthesis [88]. Pyrimidine synthesis is essential for the proliferation of activated lymphocytes, and inhibiting this pro-inflammatory pathway shifts the immune response to increasing anti-inflammatory cytokines like IL-10. Thus, teriflunomide may enhance susceptibility to TB by promoting IL-10 secretion from macrophages and microglia, which in turn suppresses antimycobacterial immunity [88]. A few cases of TB were also reported with leflunomide, an immunomodulator and immunosuppressant that inhibit synthesis of pyrimidine, with a mechanism similar to teriflunomide [89]. Another study looked into the prevalence of LTBI in MS patients by comparing TB infection in MS and neuromyelitis optica spectrum disorder (NMOSD) patients and found a frequent LTBI of 3.95% in the MS cohort [90]. This was likely due to MS therapies that suppress the immune system, promoting an opportunistic environment for TB reactivation [90].

In summary, MS has been consistently associated with an increased risk of mycobacterial infections, especially TB. Although the underlying mechanisms are not fully understood, the activation of TLRs, particularly TLR2, TLR4, and TLR9, appears to play a role in both MS and mycobacterial infections. Dysregulation of the TLR signaling pathway may weaken the immune response and promote inflammation, facilitating mycobacterial growth. Some MS treatments like teriflunomide may heighten TB risk by increasing anti-inflammatory IL-10. The relationship between MS and TB is still under investigation, and a deeper understanding of TLRs, along with their roles in MS and TB, may pave the way for future therapeutic strategies.

### 3.5. Crohn’s Disease

CD is a chronic inflammatory bowel disease characterized by transmural inflammation that can occur in any portion of the GI tract, commonly in the terminal ileum and colon [91]. The cardinal symptoms include crampy abdominal pain, non-bloody or bloody diarrhea, fatigue, and weight loss [92]. Patients with CD have an increased risk of malabsorption, malnutrition, and colorectal cancer [92]. The etiology of CD is unknown and is described as multifactorial and attributed to genetic susceptibility, environmental factors, gut microbiome, and abnormal immune response [93]. Due to the similarities in the clinical and immunopathogenic features of CD and mycobacterial infection, MAP has been proposed as the causative agent of CD [94].

MAP is an obligate intracellular bacterium known to cause Johne’s disease in ruminants [95]. Its cell wall contains 60% lipid that confers a survival advantage, acid-fast properties, and resistance to chemicals and pasteurization [96]. The distinguishing features of MAP are its extremely slow growth and its inability to produce mycobactin [97]. TNF-α and interleukins, which are involved in inflammation and tissue damage, are also encountered in CD [94].

Similarities in the clinical and pathological features of MAP and CD suggest a link between CD and increased susceptibility to mycobacterial infection [98]. Both are intestinal granulomatous diseases that preferentially target the ileum and mesenteric lymph nodes and present with segmental involvement of the intestine. They also similarly present with gastrointestinal symptoms of chronic diarrhea and weight loss and extraintestinal symptoms of amyloidosis, hepatic granulomatosis, and renal involvement [95]. Comparing the macroscopic and microscopic features, both CD and MAP result in parietal edema and lymphoid aggregates [95].

MAP in intestinal tissues and the serum markers against MAP have been detected in patients with CD at a greater frequency than those without CD. In a study conducted by Thayer et al., an enzyme-linked immunosorbent assay (ELISA) was used to examine the serum of 56 CD patients and 34 ulcerative colitis patients for antibodies against mycobacteria. Compared to the controls, patients with CD had a significant increase in antibody titer to MAP (*p* = 0.003) [98]. Bull et al. obtained intestinal mucosal biopsy samples from patients undergoing routine ileocolonoscopy for IS900 PCR testing and MAP detection. MAP was detected in 34 of 37 (92%) patients with CD and in 9 of 34 (26%) controls without CD (*p* = 0.0002, OR = 3.47). Mucosal biopsy specimens were evaluated using mycobacterial growth indicator tube (MGIT) cultures. A total of 14 of 33 (42%) MGIT culture specimens from CD patients were found to be positive for MAP compared to 3 of 33 (9%) of those of the controls (*p* = 0.0019, OR = 4.66) [99]. Clancy et al. detected cytokine secretion patterns from gut mucosal organ cultures of patients with CD, ulcerative colitis, irritable bowel syndrome, and controls. The results showed significantly higher TNF-α concentrations in MAP-positive CD than in MAP-positive UC (*p* < 0.01), MAP-positive IBS (*p* < 0.05), and MAP-positive normal controls (*p* < 0.01), respectively [100]. Since TNF-α has been found in stool as a marker of inflammation, the selective effect of MAP infection on TNF-α secretion could be a potential mechanism that could link CD and the susceptibility of MAP [100].

Genetic causes contribute to the difference in susceptibility in mycobacteria [101]. The pericentromeric region of chromosome 16 (IBD1) is most notable for several CD loci [102]. NOD2 has been mapped to chromosome 16q12. NOD2 is an intracellular receptor expressed in leukocytes and gastrointestinal tract epithelium that recognizes peptidoglycans of Gram-positive and Gram-negative bacteria and plays a role in recognizing and defending against MAP [101]. It has been shown to activate NF-kB and confer responsiveness to bacterial lipopolysaccharides (LPSs) [101]. Ogura et al. used an allele-specific PCR assay to sequence the coding exons and flanked introns of 12 CD individuals. Truncation of leucine-rich repeats (LRRs) of NOD2 was found to be associated with CD. Functional analyses revealed that mutant NOD2 is significantly less active than wild-type NOD2 in conferring responsiveness to bacterial LPS. The lack of functional NOD2 with 3020insC mutation leads to defective responses to mycobacterial peptidoglycan in Crohn’s patients, suggesting that ineffective clearance of intracellular MAP is the mechanism between MAP and CD [94]. The role of MAP and several genetic factors such as NOD2 have assisted in understanding the relationship between CD and increased susceptibility to mycobacterial infections.

### 3.6. Sjögren’s Syndrome

Beyond the autoimmune diseases discussed above, additional conditions have also demonstrated potential associations with mycobacterial exposure. Sjögren’s syndrome (SS), for example, has been increasingly linked to mycobacterial antigens through mechanisms that may involve chronic immune activation and molecular mimicry, similar to those proposed in other autoimmune diseases [103]. Altered immune responses to mycobacterial heat shock proteins (HSPs) have been observed in patients with SS, and because these proteins share structural homology with human peptides, cross-reactive immune responses may contribute to the breakdown of self-tolerance and subsequent autoantibody production characteristic of the disease [103]. Supporting this mechanistic link, a nationwide, population-based case–control study from Taiwan demonstrated a significant association between prior non-tuberculous mycobacterial (NTM) infection and the risk of newly diagnosed SS, whereas prior tuberculosis infection did not remain statistically significant after adjustment for comorbidities and bronchiectasis. Importantly, the association was strongest among individuals aged 40–65 years and those without bronchiectasis, suggesting that NTM exposure may function as an independent environmental risk factor in susceptible populations [104]. Collectively, these findings reinforce a broader conceptual framework in which chronic mycobacterial exposure may serve as an environmental trigger in genetically predisposed hosts, contributing to the development of diverse autoimmune phenotypes.

### 3.7. Mycobacterial Role in Autoimmune Disease Pathogenesis

**Figure 1:**
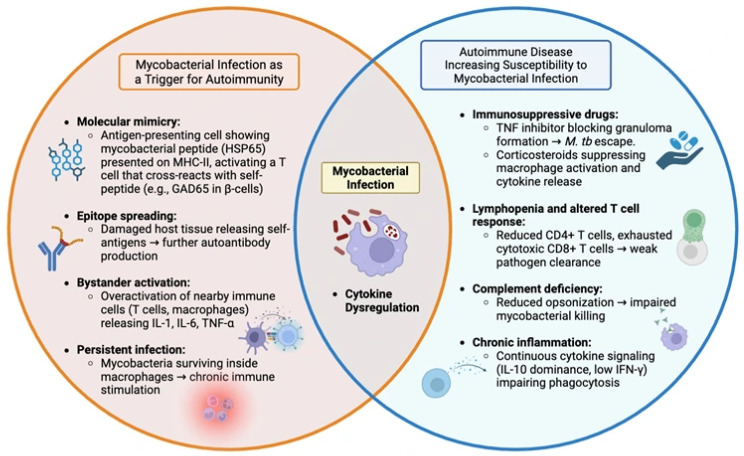
Bidirectional relationship between mycobacterial infection and autoimmunity. Mycobacterial infections may initiate autoimmune disease through molecular mimicry, bystander activation, and chronic immune stimulation, leading to loss of tolerance and autoantibody formation (left, red). Conversely, autoimmune diseases and their immunosuppressive treatments weaken host defenses, promoting *M. tb* and NTM infections (right, blue). Cytokine dysregulation (TNF-α, IL-6, IL-10, IFN-γ) and altered macrophage function sit at the intersection of both processes, highlighting the shared immune pathways linking infection and autoimmunity.

Autoimmune diseases such as RA, T1DM, SLE, and CD show increased susceptibility to mycobacterial infections. Conversely, growing evidence suggests that mycobacteria may also contribute to the development of autoimmune diseases (Figure 1). Because of structural similarities between microbial and host peptides, mycobacteria can promote autoimmune pathogenesis through mechanisms such as molecular mimicry and immune cross-reactivity involving mycobacterial HSPs.

Molecular mimicry occurs when antigenic similarities between microbial and self-peptides activate autoreactive T and B cells, resulting in autoimmune responses [105]. This mechanism has been implicated in RA, where immune reactions initially directed against mycobacterial antigens may cross-react with host joint components. For example, immunization with *M. tb* has been shown to induce arthritis due to cross-reactivity with host cartilage proteoglycans [106]. Similarly, cross-reactive polyclonal antibodies against human lactoferrin and mycobacterial antigens have been identified in RA patients [30]. TB-reactive T cells have also been observed to recognize both *M. tb* antigens and host cartilage, supporting this shared immunologic mechanism [30].

**Figure 2:**
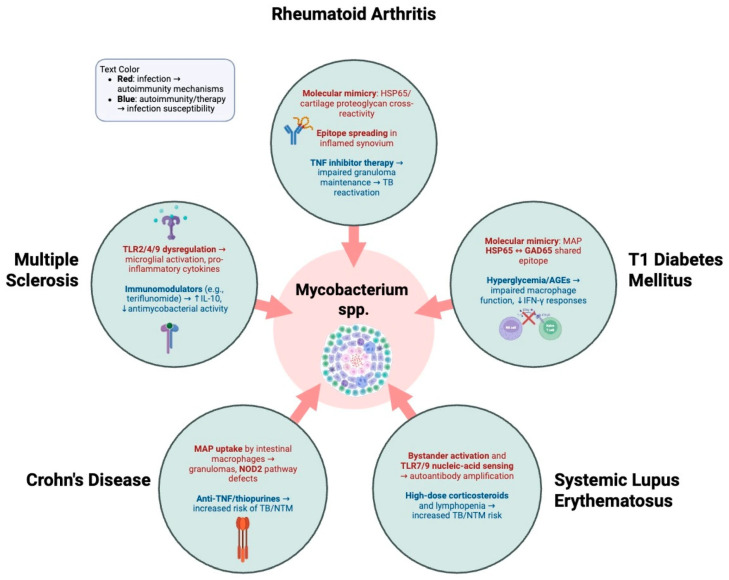
Specific mechanisms linking mycobacterial infection and autoimmune diseases. Mycobacterial infection and autoimmunity are connected by shared immune mechanisms. Red text pathways depict how microbial cues such as molecular mimicry, dysregulated TLR signaling, and cytokine imbalance may precipitate or exacerbate autoimmunity. Blue text pathways depict how autoimmune biology and immunosuppressive therapies impair antimycobacterial defenses, increasing susceptibility to TB and NTM. The red arrows point to how each major autoimmune disease discussed is linked to certain overlapping mechanisms for mycobacterial infection.

In addition, MAP has been linked to RA and T1DM (Figure 2). Bo et al. identified elevated antibody levels against two key MAP proteins, tyrosine phosphatase A and protein kinase G, in patients with RA compared to healthy controls. This finding supports a possible role of MAP-derived antigens in the loss of self-tolerance [107]. Similar mechanisms have been proposed in T1DM, where molecular mimicry between MAP peptides and pancreatic islet antigens may trigger autoimmune destruction, suggesting that chronic mycobacterial exposure could initiate or exacerbate autoimmune disease.

Multiple mycobacterium antigens, such as *M. tb*, are associated with autoimmune disorders, and one of the most prevalent antigens detected is HSP65 [30]. More specifically, the region between amino acids 180–188 can trigger autoreactive T lymphocytes, initiating a reaction with host cartilage self-proteins [108]. In patients with RA, clonal expansion of T cells against mycobacterium HSP65 due to increased responses of mononuclear cells has been detected in these patients’ synovial fluid [30]. A different study found anti-HSP65 to be elevated in patients with RA, but it was unable to find a correlation between various levels of the antibody and disease activity [109]. While it has been consistently proven that HSP65 is correlated with RA pathogenesis, interestingly, researchers have found that in rat adjuvant arthritis, HSP65 actually plays a protective role including a diversified T cell response [110]. This varied role of HSP65 and its involvement in arthritis pathogenesis remain unclear and require further investigation.

MAP HSP65 also serves as a potential source for autoimmune pathogenesis in T1DM. A study performed by Naser et al. investigated the possible role of MAP HSP65 in cross-reacting with glutamic acid decarboxylase 65 (GAD65), consequently triggering the auto-destruction of beta cells. A BLAST analysis identified a 16 amino acid region between GAD65 and MAP HSP65 with 75% similarity. Research has also suggested the same 16 amino acid region in GAD65 to be a possible antigenic binding site, causing the destruction of host beta cells [111]. Similar to how it can provide a protective effect when used in rat adjuvant arthritis, HSP65 can be used for T1DM prevention in certain contexts. Lu et al. conducted a study in which they combined a tandemly repeated portion of insulinoma antigen-2 (IA-2) with HSP65; they found that mice that were administered this fusion protein had delayed onset and lower rates of diabetes [112]. Taken together, these studies indicate that HSP65 has a nuanced and complicated role in pathogenesis and should be a topic of further research.

The similarities between host and foreign peptides have served as a potential explanation for the impact of mycobacteria on autoimmune disease. Specifically, mechanisms involving molecular mimicry and mycobacterial HSPs have demonstrated impactful roles on autoimmune pathogenesis. Although research is continuously being conducted to further explore this notion, current studies have demonstrated associations between these mechanisms and specific autoimmune diseases including RA and T1DM.

### 3.8. Other Mycobacterial Species Affecting Immunosuppression

Although *M. tb* and MAP have been the primary focus of investigation, a growing body of evidence highlights the broader relevance of non-tuberculous mycobacteria (NTM) in autoimmune populations [113,114]. Since 2000, the global prevalence of NTM pulmonary disease has steadily increased, with regional variation in dominant species, and susceptibility has been linked to rheumatoid arthritis, immunosuppressive therapy, structural lung abnormalities, genetic predisposition, and environmental factors such as warm, humid climates [113]. A large North American cohort study further demonstrated that patients receiving anti-TNF-α therapy, particularly those with rheumatoid arthritis, had markedly elevated rates of both tuberculosis and NTM disease compared with unexposed patients and the general population, with NTM infections occurring at even higher rates than tuberculosis [115]. Consistent with this expanding epidemiology, international guidelines recognize more than 190 identified NTM species, many capable of causing clinically significant pulmonary disease in immunocompromised hosts [114]. Together, these findings underscore the importance of recognizing the broad spectrum of mycobacterial pathogens when managing patients with autoimmune disease.

### 3.9. TB Vaccines and Autoimmune Conditions

Due to TB being a serious disease that requires immense attention, the Bacillus Calmette–Guerin (BCG) vaccine was developed in 1921 and continues to be widely used today to defend against childhood pulmonary TB [116]. However, its use in immunocompromised patients with autoimmune conditions remains unclear. For instance, a review article states that there appears to be a protective effect against T1DM and MS when the BCG vaccine was used; this may be due to the BCG vaccine’s ability to mitigate MAP [117]. Furthermore, intravesical BCG has been used as immunotherapy to treat urothelial carcinoma of the bladder [118]. While noted to be an effective therapy, a study found that it may be the cause of various autoimmune reactions, such as reactive arthritis, vasculitis, psoriasis, and myasthenia gravis [119]. Due to the inconsistencies found regarding the relationship of the BCG vaccine and its role in managing autoimmune disease, this is a topic that can benefit from further research.

## 4. Conclusions

Mycobacterial infection and autoimmunity form a complex, bidirectional relationship rooted in shared immune mechanisms. Mycobacterial antigens, particularly MAP and HSP65, may precipitate or amplify autoimmune responses through molecular mimicry and chronic immune stimulation. Conversely, autoimmune diseases, along with their immunomodulatory therapies, impair host defenses by disrupting T cell signaling, macrophage activity, and cytokine balance, predisposing patients to both TB and NTM infections. This reciprocal model explains observed clinical patterns across RA, T1DM, SLE, MS, and CD. Clinically, these findings highlight the need for proactive latent TB screening before initiating immunosuppressive therapy, heightened vigilance for NTM, and individualized infection risk assessment based on geography, comorbidities, and medication profile. Future research should prioritize prospective multi-region cohorts with standardized diagnostic criteria, mechanistic studies of HSP65-mediated molecular mimicry and NOD2–TLR signaling, and robust post-marketing registries to assess infection outcomes across drug classes. Investigating host-directed or immune-balancing therapies that preserve antimycobacterial immunity while mitigating autoimmunity represents a promising advancement. The integration of microbiome, genetic, and environmental data will be key to unraveling how mycobacterial exposure shapes autoimmune pathogenesis and to developing precision strategies that balance infection control with immune tolerance. Furthermore, the lack of understanding regarding the use of BCG vaccines and their impact on autoimmune conditions makes this an important topic to research further.

## Data Availability

No new data were created.

## Abbreviations

The following abbreviations are used in this manuscript:

NTM	Non-tuberculous mycobacteria
LTBI	Latent tuberculosis infection
TB	Tuberculosis
MAP	*Mycobacterium avium* subsp. *Paratuberculosis*
RA	Rheumatoid arthritis
T1DM	Type 1 diabetes mellitus
SLE	Systemic lupus erythematous
MS	Multiple sclerosis
CD	Crohn’s disease
TNF-α	Tumor Necrosis Factor- α
DMARDs	Disease-modifying anti-rheumatic drugs
TNFIs	Tumor necrosis factor inhibitors
IFN γ	Interferon gamma
HSP	Heat shock protein
GAD	Glutamic acid decarboxylase
IA-2	Insulinoma-associated protein-2
GABA	γ-amino butyric acid
TLRs	Toll-like receptors
MGIT	Mycobacterial growth indicator tube
LPS	Lipopolysaccharides
BCG	Bacillus Calmette–Guerin
